# The Importance of the Prenyl Group in the Activities of Osthole in Enhancing Bone Formation and Inhibiting Bone Resorption *In Vitro*

**DOI:** 10.1155/2014/921954

**Published:** 2014-07-24

**Authors:** Yuan-Kun Zhai, Ya-Lei Pan, Yin-Bo Niu, Chen-Rui Li, Xiang-Long Wu, Wu-Tu Fan, Ting-Li Lu, Qi-Bing Mei, Cory J. Xian

**Affiliations:** ^1^Key Laboratory for Space Bioscience and Biotechnology, College of Life Science, Northwestern Polytechnical University, Xi'an, Shaanxi 710072, China; ^2^Collaborative Innovation Center for Chinese Medicine in Qin Mountains, Xi'an, Shaanxi 710032, China; ^3^Sansom Institute for Health Research, School of Pharmacy and Medical Sciences, University of South Australia, Adelaide, SA 5001, Australia

## Abstract

Osteoporosis treatment always aimed at keeping the balance of bone formation and bone resorption. Recently, prenyl group in natural products has been proposed as an active group to enhance the osteogenesis process. Osthole has both the prenyl group and bone-protective activities, but the relationship is still unknown. In this study we found that osthole exerted a potent ability to promote proliferation and osteogenic function of rat bone marrow stromal cells and osteoblasts, including improved cell viability, alkaline phosphatase activity, enhanced secretion of collagen-I, bone morphogenetic protein-2, osteocalcin and osteopontin, stimulated mRNA expression of insulin-like growth factor-1, runt-related transcription factor-2, osterix, OPG (osteoprotegerin), RANKL (receptor activator for nuclear factor-*κ*B ligand), and the ratio of OPG/RANKL, as well as increasing the formation of mineralized nodules. However, 7-methoxycoumarin had no obvious effects. Osthole also inhibited osteoclastic bone resorption to a greater extent than 7-methoxycoumarin, as shown by a lower tartrate-resistant acid phosphatase activity and lower number and smaller area of resorption pits. Our findings demonstrate that osthole could be a potential agent to stimulate bone formation and inhibit bone resorption, and the prenyl group plays an important role in these bone-protective effects.

## 1. Introduction

Osteoporosis was first defined as a progressive systemic skeletal disease characterized by low bone mass and microarchitectural deterioration of bone tissue, with a consequent increase in bone fragility and susceptibility to fracture by the World Health Organization (WHO) expert panel in 1994 [1]. Osteoporosis is one of the most common metabolic bone diseases that often leads to osteoporotic fractures which are a major source of morbidity and mortality in the ageing population and place a significant health and economic burden on society [2]. Generally, osteoporosis is believed to be the result of imbalanced bone remodeling with decreased bone formation (carrying out by osteoblasts) and/or increased bone resorption (carried out by osteoclasts). So in the last decade, a series of new drugs have been developed to improve the bone quality, based on the understanding of the metabolism of the bone tissue, either by stimulating the bone anabolic effects, suppressing the catabolic effects, or together [3]. Although the present antiosteoporosis agents have already obtained satisfactory effects, some therapeutic drugs still possess some side effects, including the potential incidences of osteonecrosis of the jaw bone caused by large doses of bisphosphonates [4], osteosarcomas caused by high doses of PTH [5], and increased risk of endometrial and breast cancers due to prolonged use of hormone replacement therapy (HRT) [6]. Novel drugs, which could prevent the bone loss, have little adverse effects, and are cost-effective, are desired.

In recent years, Chinese herbal medicine (CHM) has attracted attention of many researchers because some of its herbs, formulations, or extracts have osteoprotective effects, low in cost, and have little or few side effects and thousands of years history of usage in osteoporosis clinical practice. Some of these herbs are* Herba Epimedii* [7],* Cnidium monnieri (L.) Cussion, Rhizoma Drynariae, *and* Psoralea corylifolia*. Among these, the dried fruit of* Cnidium monnieri (L.) Cussion*, also known as “Shechuangzi” in China and “Jashoshi” in Japan, is one of the most frequently used classic remedy for treatment of osteopenia and bone fractures [8].

**Figure 1:**
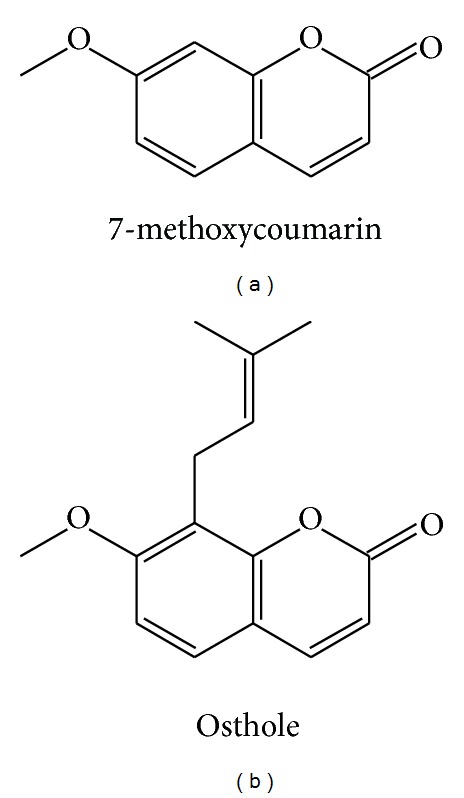
Chemical structures of 7-methoxycoumarin (a) and osthole (b).

Osthole (7-methoxy-8-isopentenoxy-coumarin, chemical structure shown in Figure 1), which is extracted from* Cnidium monnieri (L.) Cussion *and is a natural coumarin derivative, has been proved to possess many pharmacological functions such as antioxidation [9], antitumor [10], anti-inflammation [11], antidiabetes [12], and neuroprotective effects [13]. Furthermore, accumulating evidence indicates that osthole exhibits estrogen-like effects in ovariectomized rats and can reduce bone loss and prevent osteoporosis [14]. Osthole, also a prenylation coumarin compound, was found to have protective effect in the bone formation and bone resorption studies. The effects of osthole on osteoblast proliferation, differentiation [15], and osteoclast bone resorption function [16] also were confirmed. In addition, the prenyl group has been proposed as an active group to enhance the osteogenesis process in recent years of some flavonoid compounds, including genistein prenylated derivatives (6-prenylgenistein, 8-prenylgenistein and 6,8-prenylgenistein) which have been shown to better stimulate osteoblastic proliferation, differentiation, and mineralization in UMR 106 cells than genistein itself [17]. However, whether the bone-protective activity of osthole is related to the prenyl group remains unknown. In the current study, through evaluating the relationship between the osthole's prenyl group and the osteogenesis/osteoclastic resorption processes* in vitro*, we sought to address our hypothesis that the prenyl group is important for the osteoprotective property of osthole.

## 2. Materials and Methods

### 2.1. Animals and Reagents

Sprague-Dawley rats weighing 130–170 g were used for bone marrow stromal cell culture studies, new born Sprague-Dawley rats for primary calvarial osteoblast culture, and New Zealand white rabbits born within 3 days were used for osteoclast culture, which all were supplied by the Animal Center of the Fourth Military Medical University (Xi'an, China). All the procedures for cell isolation from these rats and rabbits were carried out according to the* Guide for the Care and Use of Laboratory Animals*, published by the US National Institutes of Health, and approved by Animal Ethics Committee of Fourth Military Medical University.

7-methoxycoumarin (purity > 98%) was obtained from Meryer Chemical Technology Company (Shanghai, China). Osthole (purity > 99%) was purchased from Winherb Medical Science Company (Shanghai, China). Culture media (DMEM/F12 and *α*-MEM) and fetal bovine serum (FBS) were purchased from HyClone (South Logan, Utah, USA). Penicillin, streptomycin, and trypsin-EDTA Solution were obtained from Gibco BRL (Gaithersburg, MD, USA). Majority of other chemicals/reagents were purchased from Sigma (Louis, MO, USA), including dexamethasone, *β*-glycerophosphate, DMSO (dimethyl sulfoxide), 2-Phospho-L-ascorbic acid trisodium salt, 1*α*,25-Dihydroxy vitamin D_3_, MTT (3-(4,5-dimethylthiazol-2-yl)-2,5-diphenyl tetrazolium bromide), and Alizarin Red-S and acid phosphatase stain kit. Kits for measuring alkaline phosphatase (ALP) activity and tartrate-resistant acid phosphatase (TRAP) activity were both purchased from Nanjing Jiancheng Company (Nanjing, China). Enzyme linked immunosorbent assay (ELISA) kits for the quantitative determination of osteocalcin, bone morphogenetic protein-2 (BMP-2), and osteopontin (OPN) were purchased from Cusabio Biotechnology (Wuhan, China), while the collagen-I ELISA Kit from Sengxiong Biotechnology (Shanghai, China). RNAprep pure Cell Kit for total RNA extraction, RNase-Free DNase I to remove any DNA interference, Quantscipt RT Kit for cDNA preparation and RealMasterMix (SYBR Green) for mRNA detection were obtained from TIANGEN Biotechnology (Beijing, China) and used according to the manufacturer's instructions.

### 2.2. Isolation, Culture, and Treatments of Rat Bone Marrow Stromal Cells (rBMSCs)

Three normal Sprague-Dawley male rats weighing 130–170 g were killed by cervical dislocation. Each animal's femurs and tibias were dissected aseptically, and after both ends of each bone were excised, bone marrow was collected by flushing with DMEM/F12 culture medium containing 100 U/mL penicillin, 100 *μ*g/mL streptomycin and 10% FBS with a 22 gauge needle and a syringe. Single cell suspensions were prepared by gently mixing the cells with a pipette followed by filtration through a 76 *μ*m strainer and then were cultured in 100-mm culture dishes (Nunc, Denmark) at 8 mL/dish, with an initial cell density of 5 × 10^6^ cells in 1 mL medium. Culture medium was first removed after 2 days, thereafter changed twice weekly until the cells achieved 90% confluence. At that point, cells were collected and subsequently cultured in the osteogenic medium (containing 10^−8^ M dexamethasone, 10 mM *β*-glycerophosphate, and 50 *μ*g/mL ascorbic acid phosphate) with or without 7-methoxycoumarin and osthole (dissolved in DMSO with final concentrations in culture media less than 0.05%). The osteogenic culture media were refreshed every three days and used for osteogenic differentiation assays.

### 2.3. Isolation, Culture, and Treatments of Rat Calvarial Osteoblasts (ROB)

ROB were isolated from neonatal (<3 day old) Sprague-Dawley rat calvarias by a sequential enzymatic digestion method according to Bhat et al. [18] and Hino et al. [19] with minor modifications. Briefly, eight calvariae were excised aseptically, cleaned of soft tissues, and cut into fragments (1 mm × 1 mm × 1 mm). These bone fragments were rinsed with PBS three times (each for 5 min) and then treated with 0.25% trypsin for 10 min and then digested with enzyme mixture (0.1% collagen II and 0.25% trypsin at a ratio 1 : 1) for 10 min. The first and second supernatants were discarded in order to diminish fibroblastic contamination and cell debris. Then the bone fragments were digested four times with 0.1% collagen II solution at 37°C for 20 min. Cells were obtained by centrifugation at 250 g for 5 min and cultured in *α*-modified minimum essential medium (*α*-MEM) containing 10% FBS and antibiotics (100 U/mL penicillin and 100 mg/mL streptomycin). These cells were defined as passage 0; and by 80–90% confluence they were detached by treatment with 0.25% trypsin and 1 mM EDTA and subcultured. Passage 2 cells were then seeded into 12-well plates for differentiation or 96-well plates for proliferation assays.

### 2.4. Isolation, Culture, and Treatments of Rabbit Osteoclasts (OC)

Rabbit osteoclasts were isolated by a modified procedure from a previously described method [20]. Briefly, long bones were dissected from four neonatal New Zealand white rabbits (<3 day old) and were bisected longitudinally with scissors. The inner wall of bones was scraped gently with surgical knife and flushed by injecting culture medium slowly using a sterile 25-gauge needle. Cell suspension was harvested after filtering through a 76 *μ*m cell strainer. After sedimentation for 1 min, they were seeded into 24-well plates at a density of 1 × 10^6^ cells/well on sterile glass coverslips for morphological examination or on bovine bone slices for the bone resorption measurement. After an initial culture for 12 h, medium was changed and then the cells were cultured in medium supplemented with 10% FBS, 10^−8^ M 1,25(OH)_2_D_3_, and antibiotic mixture for 9 days. The desired cultivated cells were identified as osteoclasts based on their characteristic appearance under microscopy and TRAP staining. Osteoclasts also were stained with nuclear fluorescent dyes, including hoechst 33342 (Beyotime, Haimen, China) and acridine orange (Sigma, Louis, MO, USA).

### 2.5. Proliferation Assays with rBMSCs and ROB

The proliferation of rBMSCs and ROB was determined by the MTT assay, which measures reduction of 3-(4,5-dimethylthiazol-2-yl)-2,5-diphenyltetrazolium bromide to a purple formazan product [21]. Passage 1 rBMSCs or passage 2 ROB was seeded at a density of 2 × 10^3^ cells/well in 96-well plates and cultured in DMEM/F12 or *α*-MEM supplemented with 3% FBS and 7-methoxycoumarin or osthole at different concentrations from 10^−8^ to 10^−4^ M. After 48 h, culture medium was discarded and cultures were rinsed twice with deionized water, and then 50 *μ*L MTT solution (0.5 mg/mL) was added. After incubation for 4 h, formazan crystals formed were dissolved in DMSO and the absorbance value was determined at 490 nm immediately. The effects of treatments on cell viability were expressed as ratio of Absorbance of treated cells/Absorbance of control cells [18].

### 2.6. Alkaline Phosphatase (ALP) Activity of rBMSCs and ROB

ALP activity on the 9th day after osteogenic induction culture was measured by using a colorimetric endpoint assay method [22]. Total cellular ALP activity was measured using an ALP activity assay kit and was expressed as absorbance value at 520 nm.

### 2.7. Expression of Osteogenesis-Related Genes in rBMSCs and ROB

rBMSCs and ROB were harvested and lysed at 0 h, 6 h, 12 h, 24 h, and 48 h after the osteogenic induction culture and were subjected to qRT-PCR analyses to evaluate the effects of 7-methoxycoumarin and osthole on mRNA expression of the osteogenesis-related genes, including insulin-like growth factor-1 (IGF-1), Osterix, Runx-2, OPG, and RANKL, with glyceraldehyde-3-phosphate dehydrogenase (GAPDH) as an internal control following the protocol described previously [23]. Total RNA was extracted by using RNAprep pure Cell Kit and treated with DNase I to remove any DNA contamination. The concentration and purity of total RNA were determined by absorbance at 260/280 nm in a UV spectrophotometer, and the integrity was checked by 1.5% agarose gel electrophoresis. Complementary DNA (cDNA) was synthesized by using Quantscipt RT Kit in 20 *μ*L reactions containing 1 *μ*g total RNA. qRT-PCR reaction was carried out in iQ5 PCR system (Bio-Rad, USA) by using RealMasterMix PCR Kit. Primers were designed with the Primer Express 3.0 software based on published rat cDNA sequences in NCBI and the sequences were listed in Table 1.

### 2.8. Secreted Levels of Osteocalcin, BMP-2, OPN, Collagen-I in rBMSCs, and ROB

The levels of osteocalcin, BMP-2, OPN, and collagen-I proteins produced by rBMSCs and ROB in the culture medium were determined by the ELISA Kits according to the manufacturer's instructions. Absorbance values at 450 nm of samples and standards at serial concentrations were obtained by Synergy HT Monochromator-based Multi-Mode Microplate Reader (Bio-Tek, Winooski, VT, USA) and translated into the concentrations of these proteins. Final results were expressed as ng/mL culture medium during 0–3 d, 3–6 d, 6–9 d, and 9–12 d culture periods, respectively.

### 2.9. Measurements of the Calcified Nodules Formed from rBMSCs and ROB

Calcified nodules formed at 12th day were analyzed by Alizarin Red-S staining and quantification. After rBMSCs or ROB was cultured in osteogenic medium for 12 d, cells were fixed in 4% paraformaldehyde for 10 min and subsequently washed three times with deionized water. Then the cells were stained with 0.1% Alizarin Red-S in Tris-HCl buffer (pH 8.32) for 1 h at 37°C. The stained culture plates were photographed and the areas and relative intensities of calcified nodules were quantified by using Image-Pro plus 6.0 software.

### 2.10. TRAP Staining and Activity Assay of Osteoclasts

TRAP staining of rabbit osteoclasts at 6th day of osteoclastic culture were carried using a TRAP staining kit. All the TRAP-positive cells containing three or more nuclei cells were defined as osteoclasts under a light microscope. In addition, the TRAP activity of rabbit osteoclasts at 6th day of culture was also determined by colorimetric endpoint assay using a TRAP measurement Kit and expressed as the absorbance value at 530 nm.

### 2.11. Measurement of the Bone Resorption Ability of Osteoclasts

Rabbit osteoclasts were isolated and plated on the bovine bone slices (6 × 6 mm square slices of 150–200 *μ*m thick) and then resorption pits at 9th day of culture were examined to investigate treatment effects on bone resorptive ability. Slices were fixed by 2.5% glutaraldehyde solution for 10 min and washed with 0.25 M ammonia solution for 5 min for 3 times. Then the slices were dehydrated in graded alcohols and stained with 1% toluidine blue solution for 10 min. After wash with deionized water, the resorption pits were visualized under light microscopy. Areas and relative intensities of resorption pits were analyzed using Image-Pro plus 6.0 software.

### 2.12. Statistical Analysis

The data in MTT and ALP activity assays were from six parallel replicates (*n* = 6) and other examinations from three replicates (*n* = 3). Each experiment was repeated 3 times. All data were presented as mean ± standard deviation (SD) and analyzed using one-way ANOVA followed by the LSD post hoc test using SPSS 17.0 software (Chicago, IL, USA). The statistically significant difference was considered at the level of *P* values < 0.05.

## 3. Results

### 3.1. Cell Morphological Observation of Cultured rBMSCs, ROB and OC

**Figure 2:**
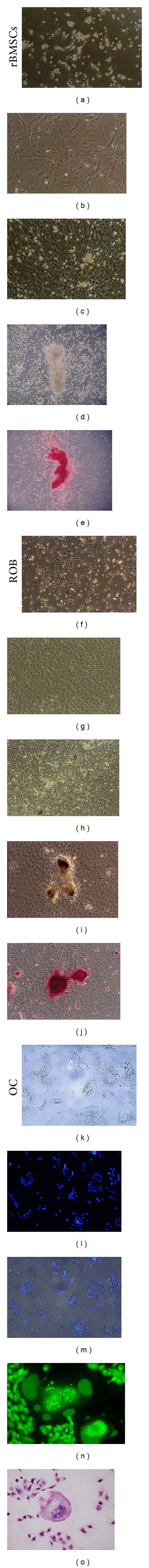
Morphology of cultured rat bone marrow stromal cells (rBMSCs), rat calverial osteoblasts (ROB), and rabbit osteoclasts (OC). Appearances of rBMSCs after being cultured for 24 h (a), after the first medium change (b), after being cultured for 8 d in osteogenic media with secreted mineral salts (c), after being cultured for 12 d showing presence of mature calcified nodules formed (d), and appearing dark red after Alizarin Red-S staining (e). Appearance of primary ROB after being seeded for 24 h (f); passage 2 cells appearing uniform and slabstone-like after being cultured in osteogenic medium 4 d (g); presence of calcified nodules after 8 d of osteogenic induction culture (h); presence of mature mineralized nodules after osteogenic induction culture 12 d (i), and appearing dark red after the Alizarin Red-S staining (j). Isolated rabbit primary osteoclasts displayed irregular shape, multinuclear, and large size (k); nuclei of osteoclasts as stained with hoechst 33342 dye (l) and the merged image (m). Multinuclear appearance of osteoclasts as displayed by acridine orange staining (n); and osteoclasts as TRAP-stained positive multinuclear cells (o). (a)–(j) were observed under the phase contrast microscopy (Olympus, 100x) and (k)–(o) were observed under Eclipse 80i Fluorescence Microscope (Nikon, 100x).

rBMSCs in the primary culture were adherent to the bottom of culture plate after 24 h culture and appeared irregular in shape (Figure 2(a)); and at the first medium change, the adherent cells were spindle-like or polygon-like (Figure 2(b)). After culture for 8 d in osteogenic media, mineral salts were seen extracellularly and the outlines of rBMSCs were not very clear (Figure 2(c)). Matured calcified nodules had formed after culture for 12 d in osteogenic media and appeared light brown before the staining (Figure 2(d)) and dark red after Alizarin Red-S staining (Figure 2(e)).

Primary ROB were mostly seen attached to the culture plate and appeared fusiform, triangle or irregular at 24 h after seeding (Figure 2(f)). Passage 2 ROB was larger in size than primary ROB and appeared uniform and slabstone-like (Figure 2(g)). After 8 d culture in osteogenic medium, passage 2 ROB secreted calcium salts and formed calcified nodules (Figure 2(h)). At 12th day, mature mineralized nodules appeared dark brown before the Alizarin Red-S stainning (Figure 2(i)) and dark red after the staining (Figure 2(j)).

Rabbit osteoclasts isolated from neonatal New Zealand white rabbits were irregular in shape, multinuclear, and larger in size than rBMSCs and ROB (Figure 2(k)). One of the most important characters of OC is the multinuclear nature, as shown in Figure 2(l) after being stained by hoechst 33342. The merged image (Figure 2(m)) of the osteoclast profiles and their nuclei showed clear locations of multiple nuclei within the osteoclasts. Multinuclear osteoclasts were also displayed in Figure 2(n) after being stained by acridine orange. Another important feature of osteoclasts is the positive TRAP-staining in the cytoplasm (Figure 2(o)).

### 3.2. Effects on Cell Viability of rBMSCs and ROB

**Figure 3:**
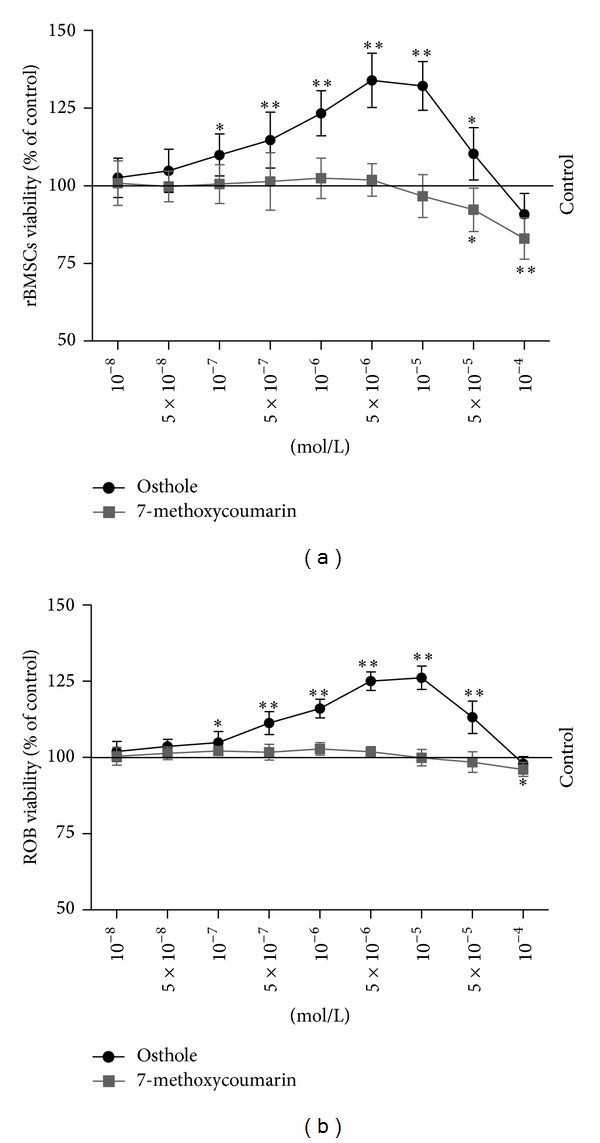
Dose-dependent effects of 7-methoxycoumarin (a) and osthole (b) on the viability of rat bone marrow stromal cells (rBMSCs) and rat calverial osteoblasts (ROB). The cells were grown and treated with culture medium containing 3% FBS and indicated concentrations of 7-methoxycoumarin and osthole. The cell viability of rBMSCs and ROB were measured by MTT assay for 48 h. The data represented three experiments and were shown as the mean ± SD of sextuplicate wells. *P < 0.05, **P < 0.01 represent significant differences between the drug-supplemented groups and the control.

Treatment effects on the viability (cell growth and survival) of rBMSCs and ROB were determined by MTT assay (Figure 3). After 48 h culture with the 7-methoxycoumarin and osthole at a range of concentrations (from 10^−8^ to 10^−4^ M), there was a significant increase in the rBMSCs viability (expressed as a ratio over the untreated control group) in the presence of osthole at a concentration range from 10^−7^ to 5 × 10^−6^ M with a dose-dependent manner (*P* < 0.05 or *P* < 0.01 versus control). While the viability of rBMSCs in osthole group declined from 10^−5^ to 10^−4^ M, the viability at 10^−5 ^M and 5 × 10^−5^ M was still higher than the control group. Although the rBMSCs viability in 7-methoxycoumarin-treated groups increased slightly at the concentration range from 10^−7^ to 5 × 10^−6^ M, the changes were not statistically significant. The viability in 7-methoxycoumarin-treated rBMSCs also decreased significantly at 5 × 10^−5^ M and 10^−4 ^M concentrations when compared with the control group (Figure 3(a)).

Osthole can stimulate the proliferation and survival of ROB as ROB viability increased in the presence of osthole at the concentration range from 10^−7^ to 5 × 10^−5^ M (*P* < 0.05 or *P* < 0.01 versus control), although the viability at 10^−4^ M was almost close to the control group. ROB viability in 7-methoxycoumarin groups was similar to the control except at the 10^−4^ M concentration at which it decreased notably (*P* < 0.05) when compared with control (Figure 3(b)).

### 3.3. Effects on ALP Activity of rBMSCs and ROB

**Figure 4:**
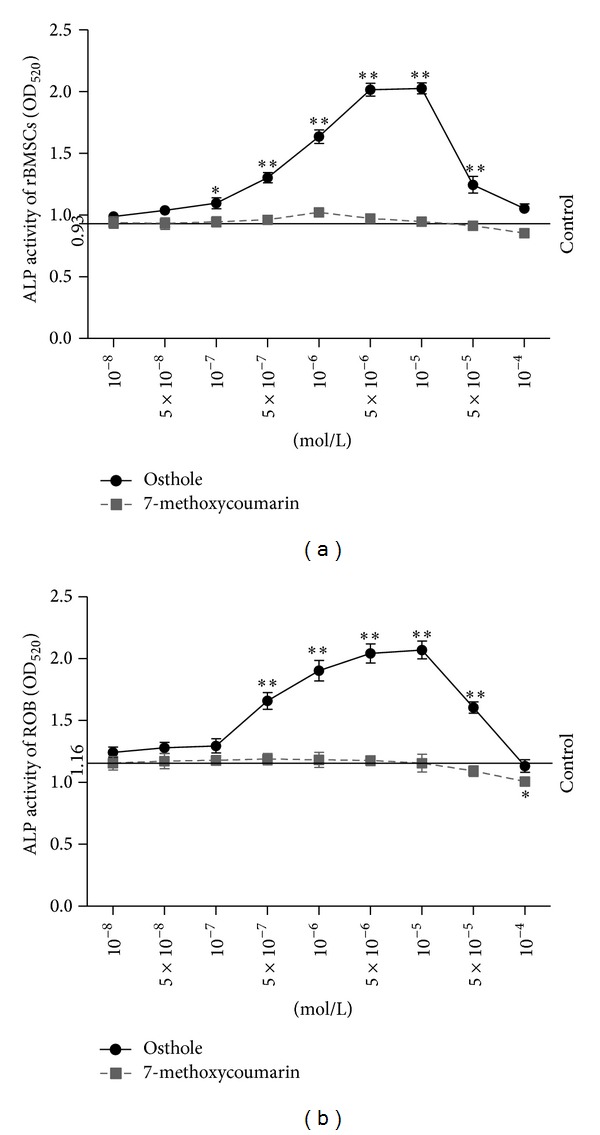
Dose-dependent effects of 7-methoxycoumarin (a) and osthole (b) on alkaline phosphatase (ALP) activity in rat bone marrow stromal cells (rBMSCs) and rat calverail osteoblasts (ROB). rBMSCs and ROB were cultured in osteogenic culture medium and supplied with different doses of 7-methoxycoumarin and osthole. Data were represented by three experiments and shown as the mean ± SD of sextuplicate wells. *P < 0.05, **P < 0.01 represent significant differences between the drug-supplemented groups and the control.

ALP activity, which indicates the osteogenic differentiation of rBMSCs and the maturation of ROB directly, was measured as a marker for screening the optimal concentration(s) of 7-methoxycoumarin and osthole in promoting osteogenic differentiation/maturation* in vitro*. As shown in Figure 4, ALP activity was increased in the presence of osthole at concentrations of 10^−8^ M to 10^−5^ M, especial at 10^−7^ M to 10^−5^ M in rBMSCs and 5 × 10^−7^ M to 10^−5^ M in ROB (*P* < 0.05 or *P* < 0.01 versus control). ALP activity in osthole-treated cells peaked at 10^−5 ^M and then declined with the increasing concentration until 10^−4^ M at which the ALP was near to the control; this tendency was observed both in rBMSCs and ROB culture systems. However, after treatments with different concentrations of 7-methoxycoumarin, there were no significant changes in ALP activity in both rBMSCs and ROB, except for a decrease at 10^−4^ M in ROB when compared with control group (*P* < 0.05). The above dose-dependent studies revealed that the optimal concentrations of osthole in rBMSCs and ROB were at 10^−5^ M and that 7-methoxycoumarin had no significant positive effects on the ALP activity in both rBMSCs and ROB. Thus osthole and 7-methoxycoumarin were used in subsequent experiments at the concentrations of 10^−5^ M.

### 3.4. Effects on the mRNA Expression of Osteogenesis-Related Genes during Osteogenic Culture of rBMSCs and ROB

**Figure 5:**
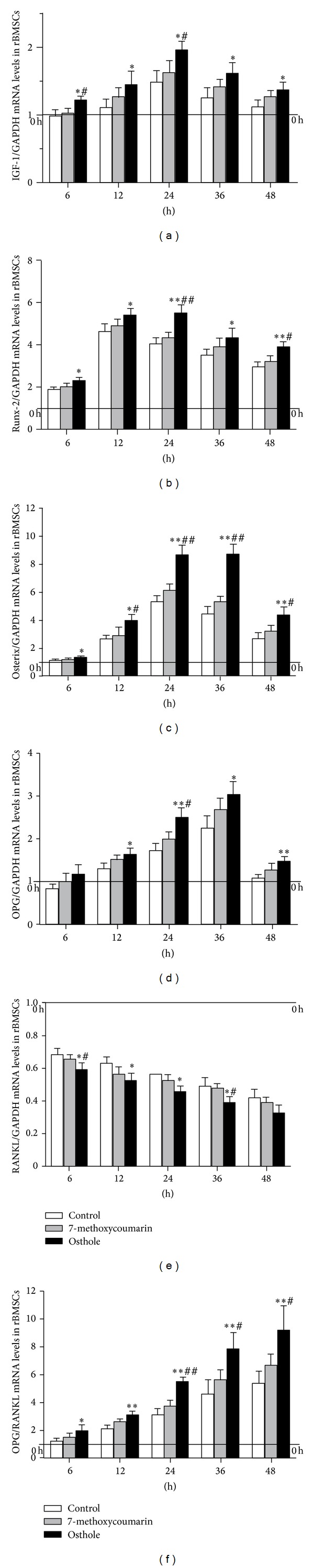
Time-course changes in mRNA levels of osteogenesis-related genes in rat bone marrow stromal cells (rBMSCs) at 6 h, 12 h, 24 h, 36 h, or 48 h after osteogenic culture treated with 10^−5^ M 7-methoxycoumarin or osthole or neither (control). Levels of expression were determined by qRT-PCR and are presented as fold changes relative to the control after being standardized against internal control GAPDH. (a) IGF-1; (b) Runx-2; (c) Osterix; (d) OPG; (e) RANKL; (f) OPG/RANKL. Results are expressed as the mean ± SD, *P < 0.05, **P < 0.01 versus control; ^#^ *P* < 0.05, ^##^ *P* < 0.01 versus 7-methoxycoumarin.

**Figure 6:**
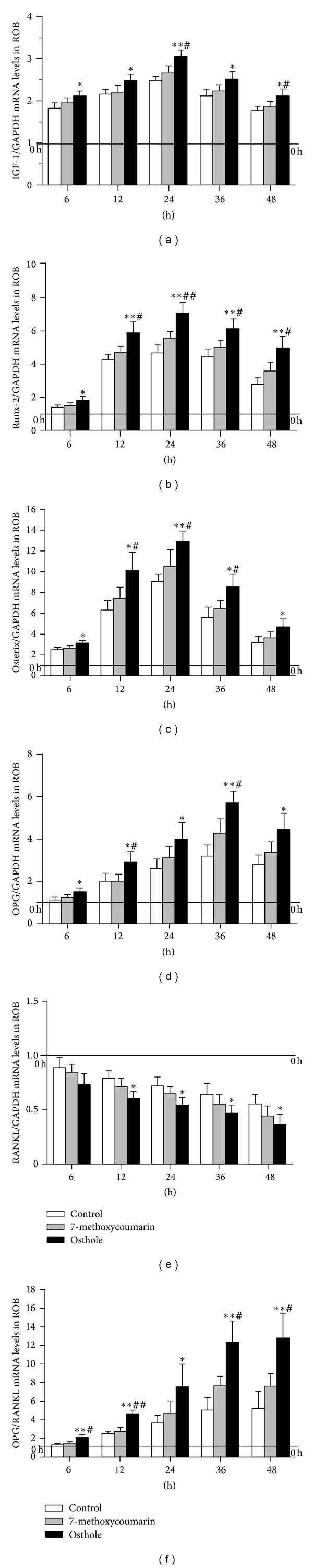
Time-course changes in mRNA levels of osteogenesis-related genes in rat calverial osteoblasts (ROB) at 6 h, 12 h, 24 h, 36 h, or 48 h after osteogenic culture treated with 10^−5^ M 7-methoxycoumarin or osthole or neither (control). Levels of expression were determined by qRT-PCR and are presented as fold changes relative to the control after being standardized against internal control GAPDH. (a) IGF-1; (b) Runx-2; (c) Osterix; (d) OPG; (e) RANKL; (f) OPG/RANKL. Results are expressed as the mean ± SD, *P < 0.05, **P < 0.01 versus control; ^#^ *P* < 0.05, ^##^ *P* < 0.01 versus 7-methoxycoumarin.

Expression of osteogenesis-related genes in rBMSCs and ROB was examined after different durations of osteogenic induction culture supplemented with 7-methoxycoumarin or osthole, including IGF-1, Runx-2, Osterix, OPG, and RANKL. IGF-1 was synthesized in the liver and enhanced the differentiated function of the osteoblast and bone formation [24].* In vitro* and* in vivo* studies have shown that IGF-1 enhances matrix apposition and decreases collagen degradation [25]. As shown in Figures 5(a) and 6(a), the mRNA expression of IGF-1 increased quickly at 6 h after osteogenic induction culture in rBMSCs and ROB, peaked at 24 h, and then declined gradually. This tendency was maintained among the control, 7-methoxycoumarin, and osthole-treated groups; however, the osthole-supplemented group always produced the highest IGF-1 mRNA level among these three groups. Although the IGF-1 mRNA levels in 7-methoxycoumarin group appeared higher than control, the differences were not statistically significant. Furthermore, the osthole group also displayed higher IGF-1 mRNA levels than 7-methoxycoumarin group, especially at 6 h and 24 h in rBMSCs (*P* < 0.05) and at 24 h and 48 h in ROB (*P* < 0.05).

Runx-2, also known as core binding factor *α*-1 (*Cbfα-1*), was thought to be the initial critical osteogenic transcription factor required for the differentiation of mesenchymal cells into preosteoblasts [26]. qRT-PCR analysis revealed that Runx-2 mRNA expression had different patterns between rBMSCs and ROB. Runx-2 levels increased with the osteogenic culture, peaked at 12 h in rBMSCs and 24 h in ROB, and then declined gradually afterwards (Figures 5(b) and 6(b)). However, Runx-2 mRNA levels in osthole group always were highest among the 3 groups in all time points examined, and in both rBMSCs and in ROB.

Osterix, an osteogenic transcription factor downstream of Runx-2, is required for the differentiation of preosteoblasts into mature osteoblasts [27]. In rBMSCs (Figure 5(c)), osthole induced a peak level of osterix after 24 h osteogenic culture and maintained a similar level at 36 h; then the osterix level decreased at 48 h. However, osterix mRNA levels in 7-methoxycoumarin and control declined slightly from 24 h to 48 h in rBMSCs and were not significantly differently between these two groups. In ROB (Figure 6(c)), levels of osterix mRNA increased and decreased synchronically in all three groups, which all peaked at 24 h. Similar with the results in rBMSCs, the osthole group still induced the highest osterix mRNA levels in ROB at all time points when compared with 7-methoxycoumarin and control groups (*P* < 0.05 or *P* < 0.01).

OPG and RANKL are two proteins secreted by osteoblasts, which critically regulate osteoclast differentiation. While RANKL stimulates the differentiation of osteoclast precursor cells into mature osteoclasts, the OPG acts as antagonist through binding to RANKL and thus inhibits osteoclastogenesis process [28]. Osthole considerably promoted expression of OPG, with its effect peaking at 36 h after the osteogenic culture and then declining slowly in both rBMSCs (Figure 5(d)) and ROB (Figure 6(d)). Furthermore, osthole group produced 6 times and 3 times more OPG than the initiation point in ROB and rBMSCs at its peak point, respectively. In contrast, RANKL mRNA levels gradually decreased with the osteogenic induction culture of rBMSCs (Figure 5(e)) and ROB (Figure 6(e)). Osthole decreased the RANKL mRNA expression most notably among the three groups in either rBMSCs or ROB culture, especially at 6 h and 36 h in rBMSCs when compared with the 7-methoxycoumarin group, although the differences between these two groups in ROB were not statistically different.

Since bone remodeling potential can also be assessed by the relative ratio of OPG to RANKL, with higher ratio values indicating stronger bone protective effects [29], effects of osthole or 7-methoxycoumarin treatments on OPG and RANKL expression in rBMSCs and ROB cultures were also expressed as OPG/RANKL ratio. With the osteogenic induction culture in both rBMSCs (Figure 5(f)) and ROB (Figure 6(f)), the ratio of OPG/RANKL increased rapidly. In rBMSCs culture, the OPG/RANKL ratios of osthole group were significantly higher than the control group from 6 h to 48 h and than the 7-methoxycoumarin group from 24 h to 48 h (*P* < 0.05 or *P* < 0.01). In ROB culture, the ratios were higher in osthole group than the control and the 7-methoxycoumarin groups at all time points except 24 h (*P* < 0.05 or *P* < 0.01).

### 3.5. Effects on the Secretion of Osteogenesis-Related Proteins in rBMSCs and ROB

**Figure 7:**
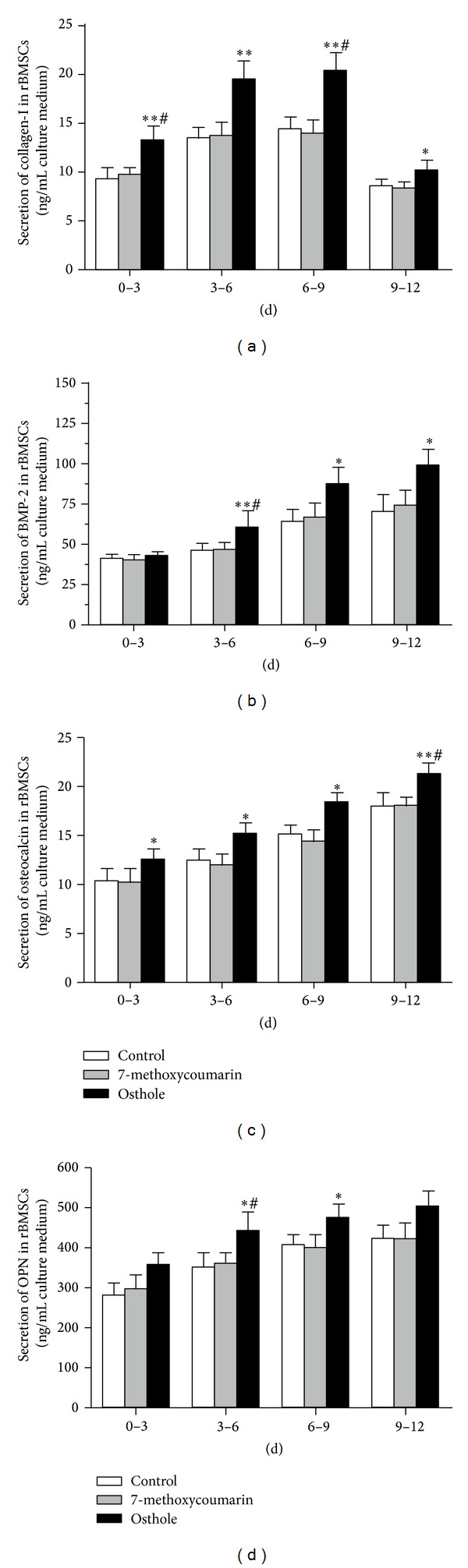
Time-course changes in the secretion of osteogenesis-related proteins after different days of osteogenic induction culture of rat bone marrow stromal cells (rBMSCs) treated with 10^−5^ M 7-methoxycoumarin or osthole or neither (control). Protein levels in conditioned medium were determined by ELISA assays for collagen-I (a), BMP-2 (b), osteocalcin (c), and OPN (d). Results were represented by three experiments and shown as the mean ± SD of triplicate wells. *P < 0.05, **P < 0.01 versus control; ^#^ *P* < 0.05, ^##^ *P* < 0.01 versus 7-methoxycoumarin.

**Figure 8:**
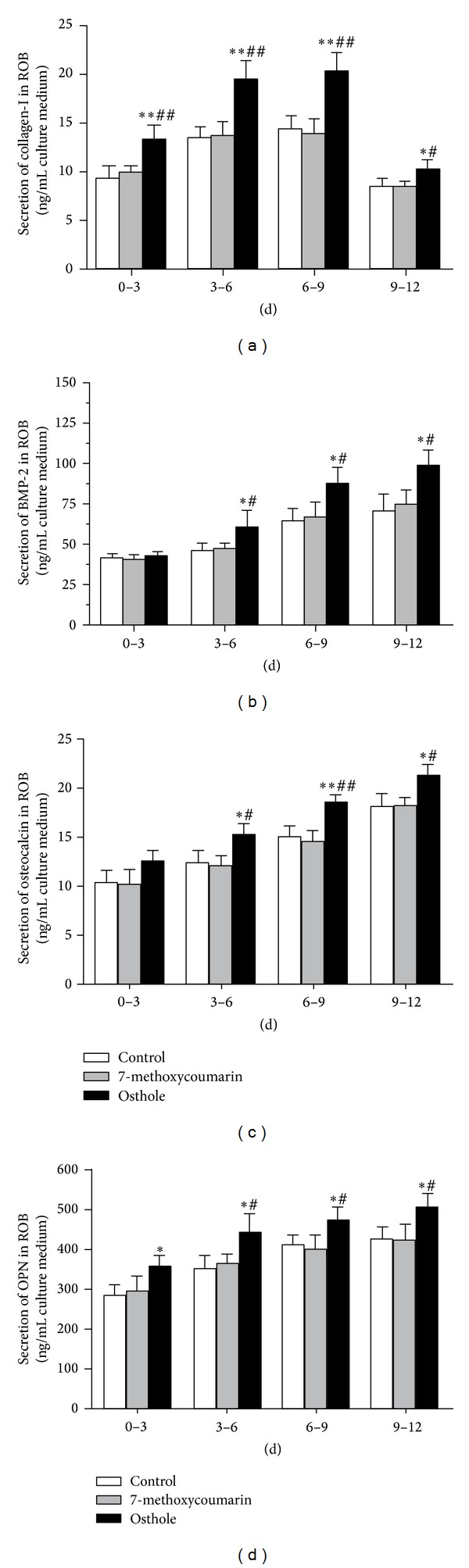
Time-course changes in the secretion of osteogenesis-related proteins after different days of osteogenic induction culture of rat calverial osteoblasts (ROB) treated with 10^−5^ M 7-methoxycoumarin or osthole or neither (control). Protein levels in conditioned medium were determined by ELISA assays for collagen-I (a), BMP-2 (b), osteocalcin (c), and OPN (d). The results were represented by three experiments and shown as the mean ± SD of triplicate wells. *P < 0.05, **P < 0.01 versus control; ^#^ *P* < 0.05, ^##^ *P* < 0.01 versus 7-methoxycoumarin.

Type-I collagen is the main component of bone matrix and its degradation always causes bone loss [30]. With osteogenic induction culture, collagen-I secretion (as indicated by the levels in the conditioned medium) increased gradually in both rBMSCs and ROB cultures, and the levels of collagen-I declined at 9–12 d time point. During the whole time course, the osthole group always induced the highest concentrations of collagen-I among these three groups. At all time points of the osteogenic culture of rBMSCs (Figure 7(a)) and ROB (Figure 8(a)), collagen-I concentrations in the osthole group were significantly higher than the other two groups (*P* < 0.05 or *P* < 0.01), while there were no differences between 7-methoxycoumarin and control groups.

BMP-2 [31] is known to induce mesenchymal cells to differentiate into osteogenic cells, and osteocalcin [32] and OPN [33], often used as the biochemical markers for bone formation, are known also to play important roles in the differentiation process of osteoblasts. As shown in Figures 7(b)–7(d) and Figures 8(b)–8(d), trends of changes of conditioned medium levels during the osteogenic culture time course are similar for BMP-2, osteocalcin, and OPN in both rBMSCs and ROB. The secretion of these proteins increased persistently with a time-dependent manner in both rBMSCs and ROB cultures. At the early stage (0–3 d), there were no significant differences in the protein concentrations among these three groups, except that osteocalcin level was higher than the control in rBMSCs (*P* < 0.05) and OPN level was higher than the control in ROB (*P* < 0.05). The secretion levels of these proteins of the osthole group were consistently higher (*P* < 0.05 or *P* < 0.01) than the other two groups during 3–12 d of osteogenic culture in both rBMSCs and ROB, with the only exception of the OPN secretion during 9–12 d in rBMSCs culture. The levels of these proteins in 7-methoxycoumarin group were not statistically different from the control levels in every time points in both rBMSCs and ROB.

### 3.6. Effect on Mineralization Potential in rBMSCs and ROB

**Figure 9:**
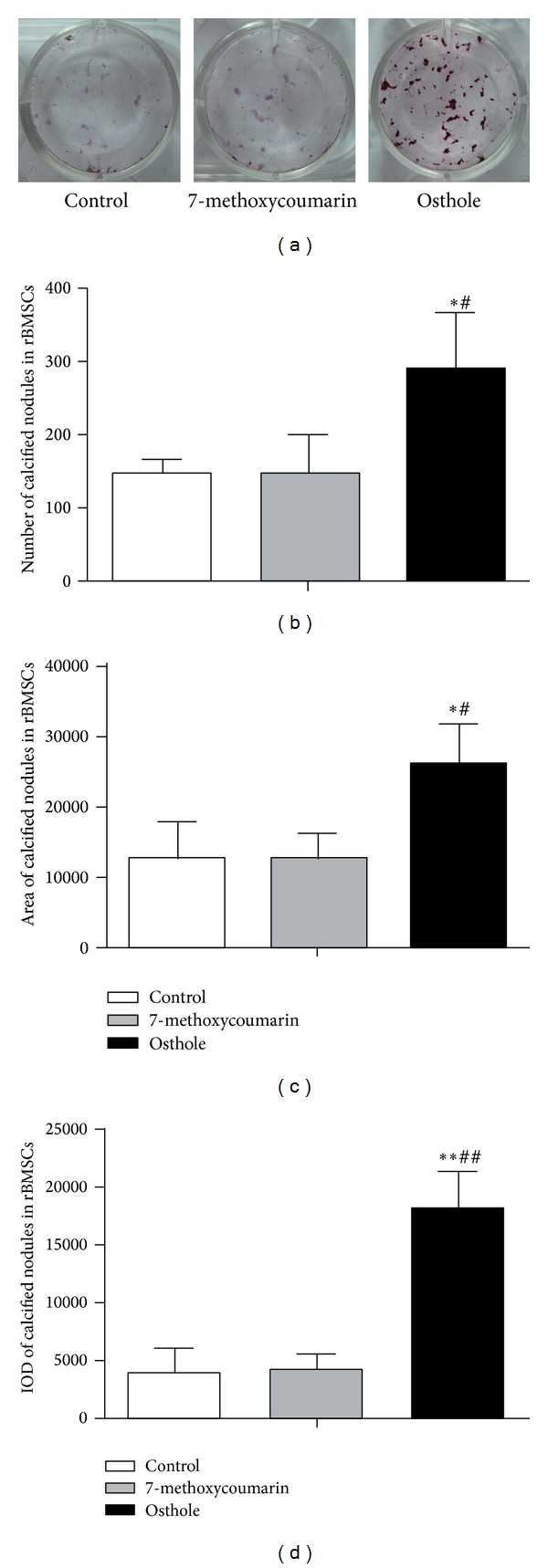
Effects of treatment with 10^−5^ M 7-methoxycoumarin or osthole or neither (control) on calcified nodules formation in rat bone marrow stromal cells (rBMSCs). Alizarin Red-S stained calcified nodules formed at 12th day after osteogenic induction culture (a). Number (b), total area (c), and staining intensity (integrated optical density or IOD) (d) of mineralized nodules were measured by Image-Pro Plus 6.0 software. The results are shown as the mean ± SD of triplicate cultures. *P < 0.05, **P < 0.01 versus control; ^#^ *P* < 0.05, ^##^ *P* < 0.01 versus 7-methoxycoumarin.

**Figure 10:**
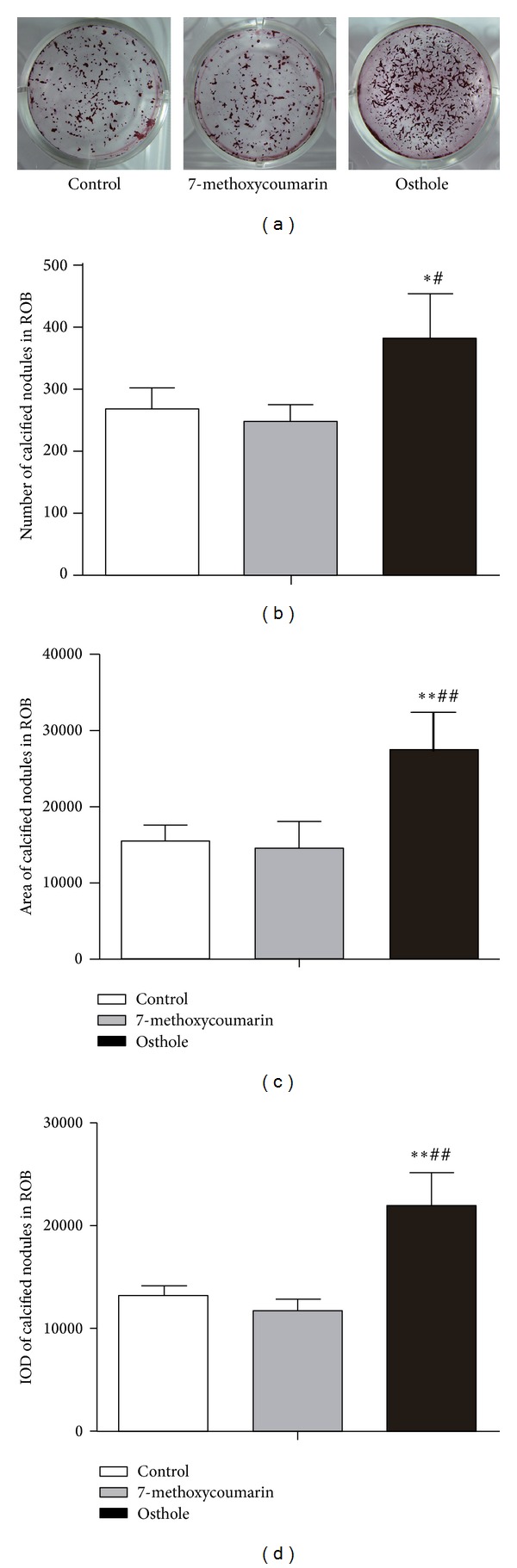
Effects of treatment with 10^−5^ M 7-methoxycoumarin or osthole or neither (control) on calcified nodules formation in rat calverial osteoblasts (ROB). Alizarin Red-S stained calcified nodules formed at 12th day after osteogenic induction culture (a). Number (b), total area (c), and staining intensity (integrated optical density or IOD) (d) of calcified nodules were measured by Image-Pro Plus 6.0 software. Results are shown as the mean ± SD of triplicate cultures. *P < 0.05, **P < 0.01 versus control; ^#^ *P* < 0.05, ^##^ *P* < 0.01 versus 7-methoxycoumarin.

To investigate the treatment effects on the mineralization potential of rBMSCs and ROB, calcified nodules formed were analyzed by staining of Alizarin Red-S at 12th day after the osteogenic culture. Among the three groups, calcified nodules in osthole group appeared larger in size, darker in staining, and more in number than the other two groups (Figures 9(a) and 10(a)), and the quantified data showed that osthole-treated group produced the highest number (Figures 9(b) and 10(b)), largest area (Figures 9(c) and 10(c)) and highest IOD (integrated optical density) staining value (Figures 9(d) and 10(d)) of mineralized nodules in both rBMSCs and ROB (*P* < 0.05 or *P* < 0.01 versus control groups), confirming the stimulatory effect of osthole in the osteogenic differentiation and mineralization. However, 7-methoxycoumarin did not enhance the formation of calcified nodules both in rBMSCs and ROB (*P* > 0.05 versus controls for all the three measurements).

### 3.7. Effects on TRAP Activity and Bone Resorption in the Rabbit Osteoclasts

**Figure 11:**
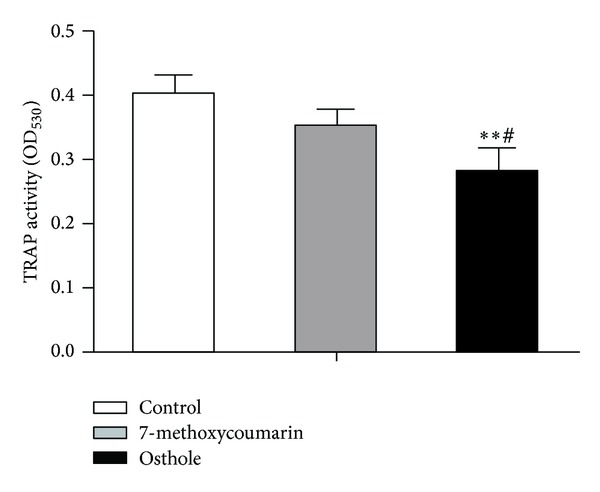
Effects of treatment with 10^−5^ M 7-methoxycoumarin or osthole or neither (control) on TRAP activity in cultured rabbit osteoclasts in *α*-MEM containing 15% FBS and 10^−8^ M 1,25(OH)_2_D_3_. TRAP activity was expressed as the absorbance value at 530 nm. Data were shown as mean ± SD of triplicate cultures. *P < 0.05, **P < 0.01 versus control; ^#^ *P* < 0.05, ^##^ *P* < 0.01 versus 7-methoxycoumarin.

**Figure 12:**
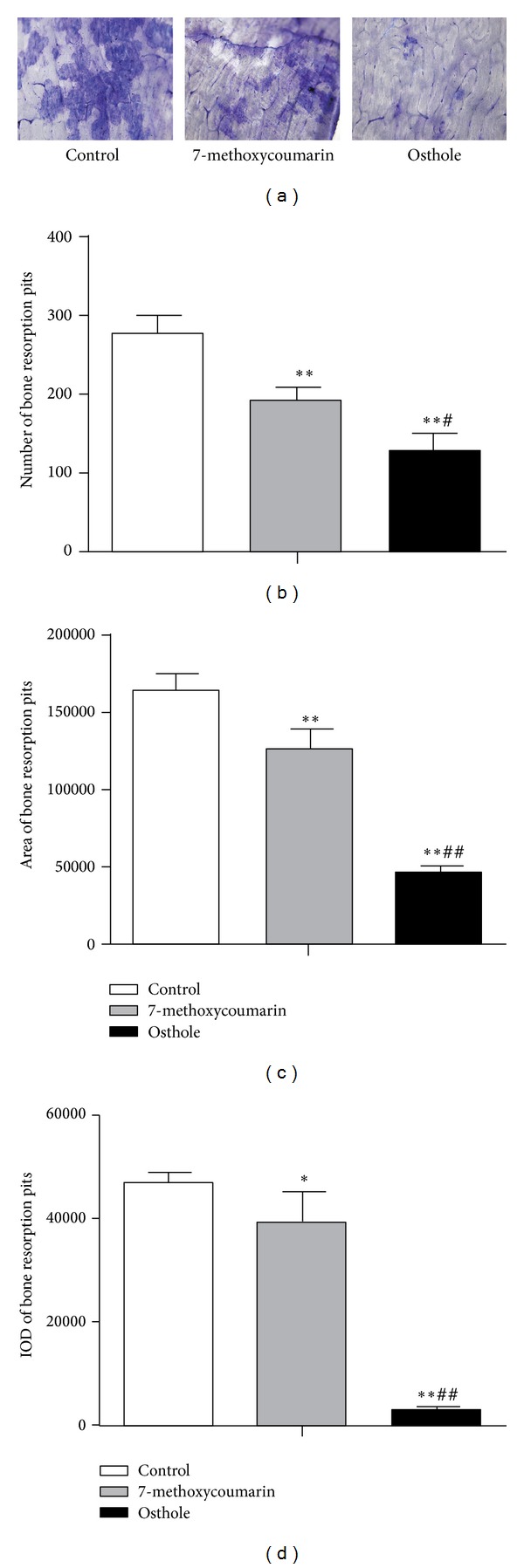
Effects of treatment with 10^−5^ M 7-methoxycoumarin or osthole or neither (control) on bone resorption pits formed by rabbit osteoclasts at the 9th day. Bone resorption pits were stained by toluidine blue (a). Number (b), total area (c), and intensity of staining (integrated optical density or IOD) (d) of bone resorption pits were measured by Image-Pro Plus 6.0 software. Data were shown as mean ± SD of triplicate cultures. *P < 0.05, **P < 0.01 versus control; ^#^ *P* < 0.05, ^##^ *P* < 0.01 versus 7-methoxycoumarin.

TRAP was one of the most important markers of the osteoclasts and was essential for osteoclast migration and bone resorption [34]. To assess treatment effects on osteoclast activity, TRAP activity was measured at 6th day of osteoclast culture. As shown in Figure 11, osthole treatment decreased the TRAP activity notably when compared with 7-methoxycoumarin group (*P* < 0.05) or with the control group (*P* < 0.01). Although 7-methoxycoumarin also had a trend of reducing the TRAP activity when compared with control, the difference did not reach statistical significance (*P* > 0.05). Consistent with the effects on TRAP activity were the effects on bone resorption activity of osteoclasts at 9th day as evaluated by the numbers, areas, and the IOD of stained pits formed by osteoclasts (Figure 12(a)). The number of pits in osthole group was lower than those in 7-methoxycoumarin (*P* < 0.05) and control groups (*P* < 0.01) (Figure 12(b)). The osthole treatment effects on the total area and IOD of pits had the same tendency as the effects on pit numbers (*P* < 0.05 or *P* < 0.01) (Figures 12(c) and 12(d)). 7-methoxycoumarin group also had a lower number, a smaller area and IOD of pits than the control group (*P* < 0.05 or *P* < 0.01) (Figures 12(b)–12(d)).

## 4. Discussion

Osteoporosis is now widely recognized to be caused by the imbalance of bone formation and bone resorption, which increases fracture risk [35]. Because of the side effects of HRT, there has been an increasing interest in looking for alternative osteoporosis treatments to the classical HRT, such as dietary estrogenic compounds (phytoestrogens) which have shown some potential benefits for osteoporosis therapy [36] and have attracted many researchers' attention [37]. Phytoestrogens comprise a set of secondary plant metabolites belonging to the chemical classes of coumestans, isoflavones, flavanones, and lignans, which all could activate the human estrogen receptor [38]. Recent studies on structure-function relationships have shown that the prenyl group may increase the estrogenic activity, even though the final activity may also depend on the position of prenyl group and the basic structure of the compound [39]. Osthole, also possessing a prenyl group, was considered to be able to prevent bone loss through an estrogen-like effect in a rat osteoporosis model study [40]. It also was shown that osthole stimulated the proliferation of osteoblast-like UMR106 cells* in vitro* [41]. However, it has been unclear how osthole exerts its potential osteoprotective effects and it has been unknown whether the prenyl group is important for the bone protective property of osthole. The current study now has also demonstrated the important role of the prenyl group in osteoprotective action of osthole in promoting osteogenesis and in suppressing osteoclastic resorption.

The current study compared the activities of osthole and 7-methoxycoumarin (differing in structure only with the lack of the prenyl group, Figure 1) in modulating the osteogenic differentiation and mineralizing function (from rat rBMSCs and osteoblasts) and formation and bone resorptive functions of rabbit osteoclasts* in vitro*. Osthole was found to stimulate rBMSCs and osteoblast proliferation, differentiation, maturation and mineralization, while 7-methoxycoumarin had no effects. In the present study, osthole was found to significantly improve the cell viability and ALP activity, enhance the secretion of collagen-I, BMP-2, osteocalcin, and OPN, stimulate the gene expression of IGF-1, Runx-2, osterix, OPG, and RANKL, and increase the number and area of mineralized nodules formed. These data strongly indicate that osthole promotes the proliferation, differentiation, and osteogenic function of rBMSCs and osteoblasts and that 7-methoxycoumarin was not shown to be osteogenic in comparison.

In addition, osthole was found to be more potent than 7-methoxycoumarin in raising the OPG/RANKL ratio in cultured rat rBMSCs and osteoblasts, which is known as an index for anti-resorption activity. Consistently, while 7-methoxycoumarin and osthole were both found to inhibit the osteoclast formation and bone resorption activity* in vitro*, osthole was found to inhibit osteoclast formation and bone resorption activity to a greater degree than 7-methoxycoumarin, as revealed by a lower TRAP activity, a lower number and a smaller area of bone resorption pits formed.

We analyzed structure-function relationships and found that the only difference in the chemical structure between 7-methoxycoumarin and osthole is the presence of the 8-prenyl group in osthole but not in 7-methoxycoumarin (Figure 1); thus any differences in stimulating osteogenic or inhibiting osteoclastic activities between the two molecules can be attributed to the 8-prenyl group. Because of this, our findings of the far greater ability of osthole than 7-methoxycoumarin in promoting osteogenic differentiation and function and inhibiting osteoclastic resorption indicated that 8-prenyl group plays an important role in the osteoprotective activity of osthole. Consistent with our argument, recently, Ming et al. compared the activities of 8-prenylnaringenin (PNG) and naringenin (NG) in osteoblast and osteoclast differentiation and their functions and showed that the 8-prenyl group played an important role in promoting osteogenesis and suppressing osteoclastogenesis and contributed to the higher bone-protective activity of PNG in comparison with NG [42].

The molecular mechanisms of the bone-protective action of osthole are still not very clear. Only two studies were found so far which related to the action mechanisms of osthole. Tang et al. [43] found that local injection of osthole significantly increased new bone formation on the surface of mouse calvaria and prevented bone loss in ovariectomized rats. They also showed that targeted deletion of the *β*-catenin and Bmp2 genes could abolished the stimulatory effects of osthole on osteoblast differentiation* in vitro*, suggesting that the stimulating effect of osthole in osteoblast differentiation may be through the activation of Wnt/*β*-catenin-BMP-2 signaling pathway. Kuo and colleagues [44] showed that osthole induces maturation and differentiation in MG-63 and hFBO cells, two human osteoblast-like cell lines. They also observed that, for osthole-mediated differentiation of osteoblast cells, BMP-2/p38 pathway activation is required for the early phase of differentiation, whereas ERK1/2 pathway activation is associated with the later phase. These two studies above indicated that the osteogenic effect of osthole involves the production of BMP-2 and may be mediated by multiple pathways and potential pathway cross-talk. Consistently, our current work also showed that osthole treatment can also strongly induced the two major osteogenic growth factors, BMP-2 and IGF-1 [24], in both rBMSCs and rat osteoblasts. Furthermore, we showed that osthole can strongly modulate production of OPG and RANKL (increasing OPG/RANKL ratio) in osteoblasts and consistently osthole was shown to potently inhibit osteoclastic resorptive function. Thus, together, data from the previous studies and our current study have now improved our mechanistic understanding for the action mechanisms of osthole in its osteoprotective (proosteogenic and antiresorptive) effects.

In summary, the present* in vitro* study proved that prenyl group mediates the beneficial effects of osthole and contributes to the higher osteoprotective activity of osthole in comparison with 7-methoxycoumarin. Although further studies are needed to explain why osthole could increase bone formation and decrease bone resorption, we at least have proved that its prenyl group plays an important role in these osteotrophic activities. While the current study and previous study [42] indicate that the addition of a prenyl group may enhance the bone-protective activity of some phytoestrogens, further studies are still needed to confirm this and to study the action mechanisms of the prenylated compounds.

## Figures and Tables

**Table 1:** Primer sequences used for qRT-PCR.

Gene	Gene bank number	Primer sequence	Product length (bp)
IGF-1	NM_001082477.2	Forward 5′-TTCAGTTCGTGTGTGGACCAAG-3′	120
		Reverse 5′-GATCACAGCTCCGGAAGCAA-3′	

Runx-2	NM_053470.2	Forward 5′-GCACCCAGCCCATAATAGA-3′	165
		Reverse 5′-TTGGAGCAAGGAGAACCC-3′	

Osterix	NM_001037632.1	Forward 5′-GCCTACTTACCCGTCTGACTTT-3′	131
		Reverse 5′-GCCCACTATTGCCAACTGC-3′	

OPG	NM_012870.2	Forward 5′-TCCTGGCACCTACCTAAA-3′	110
		Reverse 5′-ACACGCATTCATCACTCG-3′	

RANKL	NM_057149.1	Forward 5′-CATCGGGTTCCCATAAAG-3′	140
		Reverse 5′-GAAGCAAATGTTGGCGTA-3′	

GAPDH	NM_017008.3	Forward 5′-TATCGGACGCCTGGTTAC-3′	140
		Reverse 5′-CTGTGCCGTTGAACTTGC-3′	

## Abbreviations

WHO: World Health Organization
PTH: Parathyroid hormone
HRT: Hormone replacement therapy
PMO: Postmenopausal osteoporosis
CHM: Chinese herbal medicine
DMEM: Dulbecco's Modified Eagle's medium
*α*-MEM: Minimum Essential Medium Alpha Medium
DMSO: Dimethyl sulfoxide
ELISA: Enzyme linked immunosorbent assay
MTT: 3-(4,5-dimethylthiazol-2-yl)-2,5-diphenyl tetrazolium bromide
BMP-2: Bone morphogenetic protein-2
OPN: Osteopontin
rBMSCs: Rat bone marrow stromal cells
FBS: Fetal bovine serum
PBS: Phosphate buffered saline
ROB: Rat calvarial osteoblasts
OC: Osteoclasts
TRAP: Tartrate-resistant acid phosphatase
SD: Standard deviation
ALP: Alkaline phosphatase
IGF-1: Insulin-like growth factor-1
Runx-2: Runt-related transcription factor-2
OPG: Osteoprotegerin
RANKL: Receptor activator for nuclear factor-*κ*B ligand
GAPDH: Glyceraldehyde-3-phosphate dehydrogenase
Cbf*α*-1: Core binding factor *α*-1
IOD: Integrated optical density
PNG: 8-prenylnaringenin
NG: Naringenin.
